# Clinical Remarks upon Surgical Cases Occurring in the Buffalo Hospital of the Sisters of Charity

**Published:** 1875-01

**Authors:** Julius F. Miner


ART. II.—*Clinical Remarks upon Surgical Cases Occurring at the Buffalo Hospital of the Sisters of Charity.* By Prof. JULIUS F. MINER, M. D. Reported by W. W. MINER, M. D.

CASE XII. *Fracture of the Fibula.*—We have now in the hospital, four cases of fractured fibula, which you have seen. In two of them there was some severity of injury to the soft parts, which caused an amount of swelling, ecchymosis, slight puncture of integument at point of fracture, while in the others the fracture of the bone alone was specially noticeable. Of the simple, compound and comminuted varieties of fractures, which occur, the compound has been considered greatly more serious than the others, but its importance has been exaggerated. I do not regard a compound fracture as such, specially serious; it is now and then accompanied by the more severe conditions of injury, but not necessarily. The tibia in these cases here, is not broken, but serves as a splint, while repair of the fibula takes place; and in other cases where a sound bone thus supports a broken one, the necessity of applying an external splint is much smaller than usual, sometimes an external splint in such cases is altogether unnecessary. In fact I have successfully treated the most serious cases of fracture, accompanied by loss of muscular structure, without any splint whatever. A pillow alone is frequently the best receptacle and splint for a broken leg. In using such soft material especially, you need to keep track of the condition in which the bones are, and in general also, you should know how the bones are doing, even when various firm external appliances, suitable for like cases are employed. If you know that the bones are in proper position, it may not be necessary to remove the dressings till repair is completed; the necessary thing is to know that everything is right, whatever you use or do. You need to be very careful not to apply the dressings too closely, soon after an injury, before the swelling which follows, has reached its height. You will probably do more harm surgically the first year of your practice, in applying tight dressings, than in any other way. In one of the cases here, more swelling than is usual took place, and the dressings became very tight and painful, before arrival and examination at the hospital; several large blisters, filled with serum which had exuded from the congested capillaries, were found on removing the bandages. You have noticed remarkable discoloration in the whole leg in one instance; this is not of importance here, it often occurs where the injury received was one of the slightest that ever produce fracture. It is the coloring matter from minute extravasations of blood from the capillaries, it disappears with the repair of the bone. Amputation by a sloughing process is sometimes unexpectedly performed with too tight bandages. I have saved extremities sometimes by slitting the integument with a knife for the whole length of a bone, where contusion had produced swelling so great that the integument restricted the dilatation of vessels, and circulation in them, so as to strangulate the limb, acting in the same manner as tight bandaging does. One of the cases here, came in dressed with side splints of wood on both sides of the leg, one with a side splint on the outside, one with pasteboard splints, and you have seen a starch bandage on a patient elsewhere. In one of these cases here, I recommended no splint, on the others there has been used a side splint on the inside, padded so that when the bandage is applied, the ankle is brought around so as to turn slightly inwards, in order, if possible, to counteract the natural tendency of the foot in these cases to be turned outwards after repair of the fracture. Three weeks generally is time enough for bony union, and as long as splints need be used. The results here, are that one turns noticeably outwards, another scarcely so, while this tendency would hardly be detected in the others. The generally obtained result is an outward inclination of the foot, and the ankle is rendered permanently less firm than before injury, though these are both of not great importance as far as ordinary use of the limb is concerned. The rupture of the ligaments of the joint, in addition to the fracture may be the cause of this, the foot may not have been kept in suitable position to ensure union of the fibula in perfect position; the causes I am not so sure of, but the general result is what I have stated.

Case XIII. *Epithelioma of Lip.*—This old gentleman has what is sometimes called smoker's cancer, on his lower lip; an epithelial cancer is the usual term for this affection. It is a kind of malignant growth which has less tendency to recurrence after removal than do other cancerous growths. Its appearance is often supposed to be occasioned by the irritation of the parts, from using a pipe, or other means of irritation, but there seems little ground for belief in such causation. This is certainly now an eminently proper growth for excision. This is done, removing a V shaped piece of the lip, and bringing the parts together with the twisted suture. The mouth seems to you constricted to a great degree, on the closure of the wound, but this will remedy itself after a while; large portions may be removed, and the oral orifice will dilate enough to compensate for it after a little.

Case XIV. *Bubo, etc.*—This young man had when entering the hospital a bubo in his left groin, also a sore throat and mouth. He had been for sometime under treatment at one of the charitable institutions in this city, and says that he did have a running from his penis when the swelling began in his groin, and has had no other troubles. It might be supposed that this fellow has a syphilitic bubo and sore throat; this would not be a mistake hard to make, but what ground would such conclusion rest on. If you are experienced, you may with me recognize a characteristic smell here, and it is not that of syphilis. The fellow spits a great deal, and very frequently, his gums are red and swollen, his pulse fast, his mouth so sore you cannot look at his throat, to see whether it is a syphilitic sore throat or not. The fact is, he has been taking mercury to quite an extent, and has a condition called mercurial fever.

We will order a discontinuance of all medicine and see the result. His gonorrhœa has ceased, the bubo is to be opened, when the proper time arrives, and is poulticed meanwhile. Buboes arise from gonorrhœa, syphilis, chancroid, or from the slightest non-specific causes. Many are in the habit of considering all buboes specific, and hence mercury has here been given for a gonorrhœal bubo. In opening buboes, it is much better to puncture them with a small knife, and then cut from within outwards; it causes less pain, is a safe and good method. The case is a very instructive one, and may be of service to you. After a short time in bed, the secretion of saliva became natural, swelling of glands and the sore throat vanished. The bubo continued discharging sometime after it was opened, though it has now ceased.

CASE. XV. *Fracture of Tibia and Fibula.*—This good woman, a few days since, met with an accident that disabled her left leg. The nature of the injury can be seen at once by a skillful surgeon, with very little manipulation, simply raising the foot by the toes is enough to point out the nature and extent of fracture. Both bones of the leg are fractured at a point about three inches above the ankle joint. There is some spasmodic twitching of muscles, which is always present in connection with a broken bone, and this is especially troublesome at night, when it wakes the patient out of a sound sleep. It continues sometimes until union of the fracture has taken place. In dressing fractures you need never be at a loss for splints to do it with. I never was in a shanty where material could not be found for a splint. In cases where only one bone of two which are side by side, is broken, you will not need to exercise the care in adapting the splint that you do here. It is desirable that the two side splints used, should be adapted nicely to the limb when applied, so that the bones be brought, and kept in proper position. With wadding or by proper padding, the splint may be made to conform closely to the particular case. By placing the leg after the side splints are applied, in a pillow, it is left in a very comfortable position for the patient, and by this means it can, without fear, be moved by the friends, as may be necessary. Patients are often rudely carried after injury, without being put in any sort of shape for transportation; a broken leg has sometimes been left to dangle over the end of a wagon. In the use of splints the pressure on the limb should be evenly adjusted, else pain, and perhaps sloughing may result. There is a shortening which results in oblique fracture of the tibia and fibula, which varies from half to three-fourths of an inch in general. One-half an inch shortening is a good result, and there is no method of treatment of a fractured leg that entirely prevents shortening. I do not often make use of extension in the leg; do in the thigh. Very few cases of fracture below the knee are to be benefited by its use. Very numerous are the materials or appliances used for splints, such as wood, gutta percha, sized felt, plaster, silicate solutions, starch, etc, and these are applied as anterior, posterior, or lateral splints, or are made to entirely envelop the extremity. In some cases where a suppurating surface is present, any closely applied splint may not be practicable, or can be used only over a particular region. Again, sometimes the swollen condition of the soft parts, forbids any obstruction to capillary circulation, by close splints or bandaging, and a pillow alone, is the best and most satisfactory means for treatment. There is no doubt I have saved many limbs in this way, that any close dressing would destroy.

